# A New Era of Morphological Investigations: Reviewing Methods for Comparative Anatomical Studies

**DOI:** 10.1093/iob/obad008

**Published:** 2023-03-17

**Authors:** K L Ford, J S Albert, A P Summers, B P Hedrick, E R Schachner, A S Jones, K Evans, P Chakrabarty

**Affiliations:** Department of Biological Sciences, George Washington University, Washington DC, USA; EAWAG Aquatic Research Institute, Luzern, Switzerland; Institute of Ecology and Evolution, Universität Bern, Bern, Switzerland; Department of Biology, University of Louisiana at Lafayette, Louisiana, USA; Department of Biology, Friday Harbor Labs, University of Washington, Washington, USA; Department of Biomedical Sciences, College of Veterinary Medicine, Cornell University, Ithaca, NY 14853, USA; Department of Cell Biology & Anatomy, School of Medicine, Louisiana State University Health Sciences Center, Louisiana, USA; Museum of Natural Science, Louisiana State University, Louisiana, USA; BioSciences, Rice University, Houston, TX, USA; Museum of Natural Science, Louisiana State University, Louisiana, USA; American Museum of Natural History, New York, NY, USA; Smithsonian Institution, National Museum of Natural History, Washington DC, USA; Canadian Museum of Nature, Ottowa, Ontario, Canada

## Abstract

The increased use of imaging technology in biological research has drastically altered morphological studies in recent decades and allowed for the preservation of important collection specimens alongside detailed visualization of bony and soft-tissue structures. Despite the benefits associated with these newer imaging techniques, there remains a need for more “traditional” methods of morphological examination in many comparative studies. In this paper, we describe the costs and benefits of the various methods of visualizing, examining, and comparing morphological structures. There are significant differences not only in the costs associated with these different methods (monetary, time, equipment, and software), but also in the degree to which specimens are destroyed. We argue not for any one particular method over another in morphological studies, but instead suggest a combination of methods is useful not only for breadth of visualization, but also for the financial and time constraints often imposed on early-career research scientists.

## Introduction

In his landmark book entitled *On Growth and Form*, D'Arcy Thompson (1917) wrote: “We begin by describing the shape of an object in the simple words of common speech: we end by defining it in the precise language of mathematics; and the one method tends to follow the other in strict scientific order and historical continuity.” We see this progression in the explosion of studies using increasingly accessible (and ever-improving) imaging technology such as CT (computed tomography) and MRI (magnetic resonance imaging), which can be used to more precisely document and quantitatively analyze morphology with high accuracy and precision. In addition to this added rigor and accessibility, digital scanning technologies also allow for in-depth qualitative anatomical comparisons and need not be quantitative. Such technologies have been widely embraced by comparative morphologists and are granting us new insight into organismal form and function across a wide range of species.

Modern morphological analyses are based primarily on the use of several key techniques: descriptive or qualitative data from gross dissections and histology, quantitative data from linear and geometric morphometrics, and digital scanning. Traditional morphological analysis (defined here as all those not related to digital scanning) has been used for descriptive work since before the time of Aristotle, which includes two-dimensional (2D) illustrations (e.g., line drawings and photographs) of three-dimensional (3D) features or written descriptions about traits often expressed in telegraphic language (e.g., “lower jaw profile straight. ... posterior margin of maxilla exposed and reaching a vertical through anterior part of orbit”; Chakrabarty et al. 2010). Such descriptions are immensely valuable and have long been paired with linear morphometric analyses allowing quantitative comparisons among taxa (e.g., Gould 1966). However, descriptive, photographic, and linear morphometric comparisons can suffer from the fact that they can neglect precise 3D information regarding shape, position, orientation, and composition (e.g., bone surface texture and internal structure). Anatomical information in three dimensions often provides unique insights into form and function, providing critical data used in identifying homologous structures and morphometric landmarks (e.g., Evans et al. 2019; Ford and Albert 2022; Ford et al. 2022), and in assessing the biomechanical roles of these structures. Generating 3D models of biological specimens enhances all types of comparative analyses, whether descriptive, morphometric, or functional (e.g., Sherratt et al. 2014). Animals are also active, mobile creatures (and their dead tissues are not); therefore scanning techniques may not allow for the investigation of biologically-relevant movements that can be examined using clearing and staining methods (although see XROMM [X-ray Reconstruction of Moving Morphology]; Taylor 1967; Brainerd et al. 2010; Brainerd et al. 2016; Camp et al. 2020).

**Fig. 1:**
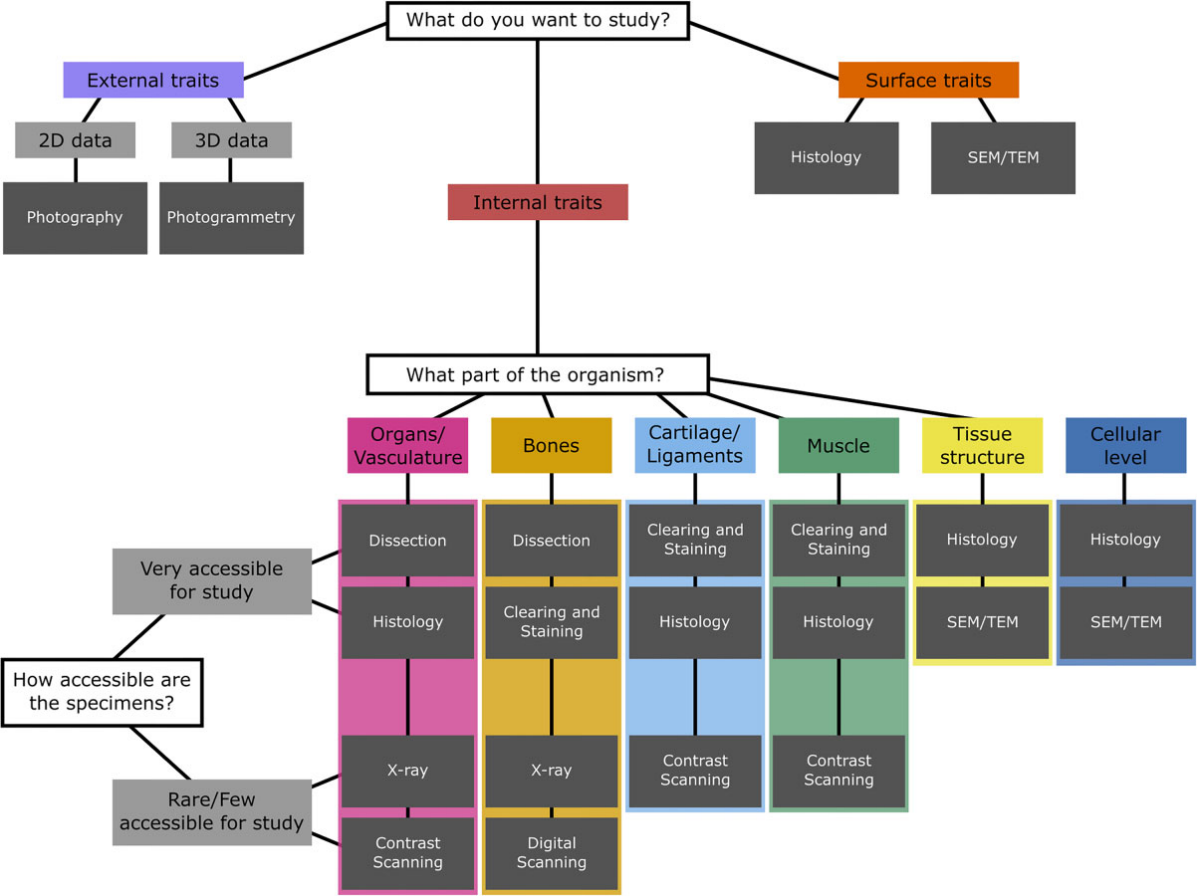
A flow chart for researchers to determine the best methods for their morphological studies; the chart showcases nine methods for examining external, surface, and internal traits, and inquires about specimen availability as it relates to destructive sampling.

Here we compare and contrast the scientific value of several morphological techniques, paying attention to the cost, benefits, and limitations of each, with a focus on how complementary techniques can be used to generate accurate and rigorous results. Costs come in several forms: financial, specimen degradation and destruction, training time, implementation and dissemination, software, and data accessibility (Table 1). Each method has associated costs which, depending on the research question, may be outweighed by several key benefits. As scanning techniques are the newest methods for visualizing morphology and complex structures, we review these methods in greater detail than the better-known traditional techniques. Specifically, we begin by discussing general assessments of specimens (e.g., photography and simple external measurements), discuss methods for generating data from rare specimens for which the potential of destructive sampling is limited or impossible (photogrammetry, X-ray, CT scanning, contrast-enhanced CT scanning, and MRI), and then discuss methods for which destructive sampling is possible (dissection, clearing and staining, histology, scanning electron microscopy [SEM], and transmission electron microscopy [TEM]). We close with commonly employed techniques for collecting data using this plethora of techniques such as qualitative descriptions, linear morphometrics, as well as 2D and 3D geometric morphometrics. Our goal with this paper is to showcase available methods for morphological studies and provide insight for early-career researchers (or to those new to morphological investigations) about which methods to use in their research investigations (Fig. 1).

**Table 1:** Comparisons between methods in comparative morphology, including photography, dissection, photogrammetry, X-ray, clearing and staining, histology, SEM/TEM, digital scanning, contrast digital scanning, and MRI

Method	**Photography**	**Photogrammetry + surface scanning**	**Dissection**	**Clearing & staining**	**Histology**	**SEM/TEM**	**X-ray**	**Digital scanning**	**Contrast digital scanning + MRI**
**Used to study:**	External + internal traits	External traits	Internal traits	Internal traits	Internal traits + surface traits	Internal traits + surface traits	Internal traits	Internal traits	Internal traits
**Data output:**	2D photographs and/or illustrations	3D files	2D photographs and/or illustrations	2D photographs	2D photographs and/or illustrations	2D photographs and/or illustrations	2D photographs and/or illustrations	3D files	3D files
**Effect on specimens:**	Nondestructive	Nondestructive	Destructive	Destructive	Destructive	Destructive	Nondestructive	Nondestructive	Nondestructive
**Data availability:**	Files must be digitized (with user error) for subsequent analyses	Digital files that can be shared and used in subsequent analyses without user error	Files must be digitized (with user error) for subsequent analyses	Must be digitized (with user error) for subsequent analyses, or physical specimens	Must be digitized (with user error) for subsequent analyses, or physical specimens	Must be digitized (with user error) for subsequent analyses, or physical specimens	Files must be digitized (with user error) for subsequent analyses	Digital files that can be shared and used in subsequent analyses without user error	Digital files that can be shared and used in subsequent analyses without user error
**Considerations:**	Useful for rare specimens	Useful for rare specimens	Often best for easily accessible specimens	Often best for easily accessible specimens	Often best for easily accessible specimens	Often best for easily accessible specimens	Useful for rare specimens	Useful for rare specimens	Useful for rare specimens
**Types of analysis:**									
Discrete characters	X	X	X	X	X	X	X	X	X
Linear measurements	X	X	X	X			X	X	X
2D geometric morphometrics	X			X			X		
3D geometric morphometrics		X						X	X
Internal homology assessment			X	X	X	X	X	X	X
External homology assessment	X	X							
Density + volume + surface area measures							X	X	X
**Time and skill**									
Specimen preparation			X	X	X	X			X
Technique training	X	X	X	X	X	X	X	X	X
Anatomy training	X	X	X	X	X	X	X	X	X
Software training		X						X	X
Measurement training	X	X	X	X			X	X	X
Interpretation training	X	X	X	X	X	X	X	X	X
**Equipment needed:**									
Software (0–$$$)		X					X	X	X
Chemicals ($)		X		X	X	X			X
Dissection tools ($)		X	X	X	X	X			
Camera ($$)	X	X	X	X	X	X	X		
Microscope ($$)	X		X	X	X	X			
Computer ($$–$$$)	X	X		X	X	X	X	X	X
Table X-ray ($$)							X		
Surface scanner ($$$)		X							
Microtome ($$$)					X				
SEM/TEM ($$$$)						X			
CT scanner ($$$$)								X	X

Abbreviations: 2D, two-dimensional; 3D, three-dimensional; CT, computed tomography; MRI ,magnetic resonance imaging; SEM, scanning electron microscopy; TEM, transmission electron microscopy.

## Focus of study: external traits

### External physical examination and photography/illustration

Physical examination of voucher specimens and the photography/illustration of those organisms offer the opportunity to study the external characteristics of organisms. One can perform qualitative examinations, morphological descriptions, linear morphometrics, and geometric morphometrics using digital 2D photographs. Such methods are particularly advantageous when studying rare or important specimens that cannot be destroyed or dissected.

#### Benefits

Cameras and digital calipers can cost on the order of hundreds of dollars, making them much less expensive than imaging equipment used to generate 3D models; especially models that include aspects of internal structure. While taking linear measurements of soft tissues (e.g., of muscles or nerves) can damage specimens, these methods generally are less damaging than histology.

#### Downsides

A substantial cost for these methods is the time, price, and carbon footprint of traveling to multiple natural history collections to collect specimen data if no digitized data exist, or shipping costs to send specimens to locations for analysis. There is also reduced resolution in the data compared to data collected using 3D modalities. In studies utilizing 2D geometric morphometrics, shape files (often .tps files) are commonly published in the supplemental information of papers. However, field standards do not require the underlying images to also be published, and there is no centralized repository to do so. To ensure that shape data are captured accurately, all specimens in an analysis must be photographed in a well-defined way to ensure that differences in resulting landmark configurations are differences in shape between specimens rather than differences in the orientation of the specimen at the time it was photographed (i.e., how was the specimen oriented relative to the lens). As a result, it is difficult to combine images or shape files taken by one researcher with those taken by another without establishing low inter-observer error in specimen photography. As a result, it can be difficult to build on previous 2D geometric morphometrics analyses.

### Photogrammetry

Photogrammetry uses digital images taken from multiple angles to construct a 3D model of a physical object (Kano 2022). Photogrammetry is often used to measure external characteristics, although it can be combined with invasive techniques (e.g., dissections, clearing, and staining) to create models of internal traits.

#### Benefits

This method allows for visualizing the color, size, volume, surface area, and shape of objects. Photogrammetry is much more cost effective than high-resolution scanning methods; the main costs include any digital camera, even the camera built into a mobile phone, and software on a computer to perform 3D reconstruction and downstream analyses. This method can be combined with other methods to examine internal traits as well, although that is typically destructive. A major benefit is the ability to readily share digital 3D objects with other researchers through open-source frameworks. Photogrammetry is still being developed for additional uses and improved results, but an ever increasing number of scientific studies are using this technique due to its reduced costs (e.g., Deakos 2010; Giacomini et al. 2019).

#### Downsides

Photogrammetry is highly dependent on the quality of the camera used, and the resulting 3D object is often not at the same resolution as other 3D visualization methods (i.e., CT scanning). A powerful computer with standard video-gaming specifications (microchip speed and a graphics card) is needed to stitch the hundreds of 2D digital images into a 3D model. Training is required to capture and analyze images, learn the reconstruction software and downstream analyses (measurement methods and interpretation). Additionally, the specimens must be in hand for this method, meaning either travel or shipping is required to obtain the data.

## Focus of study: internal traits

### If specimens are rare (limitations on specimen number)

In many cases, natural history collections may consider the loss or degradation of a specimen via invasive or destructive dissections or histology to be too high when compared with the long-term research value of the specimen, and not permit destructive sampling. The value of the research output from such specimens may be limited because the number of samples (and species) represented is smaller than ideal (or necessary) to answer the target research question. The collections may allow some (nondestructive) methods to be used to examine the morphology of organisms that are considered rare or important.

### X-ray

Radiographs are a noninvasive technique that can be used to view internal skeletal and soft-tissue anatomy and take measurements in 2D (Hedrick et al. 2019a; Cobb and Sellers 2020).

#### Benefits

X-ray machines are less expensive than other digital scanners and can provide much of the same information about skeletal arrangement. Time needed to complete 2D X-rays is much lower than that needed for 3D scans; orders of magnitude more X-rays can be completed in the same timeframe. No special software is required for analyzing these data; photographs and open-source software for measurements can be used for these studies (e.g., Fiji/ImageJ). This is helpful for students and early-career researchers who want to examine high volumes of skeletal data at rapid rates and low cost.

#### Downsides

The compression of 3D features into a flat 2D image reduces the resolution of morphometric data. Further, radiographic equipment is expensive to purchase and maintain, though not on the order of CT or MRI scanners. Additionally, training is required to learn the techniques of this approach and to understand the anatomy for correct measurements or comparisons. These data (usually as 2D photographs or illustrations) can be used in publications and for reference, but cannot always be added to another dataset (particularly of a different type) for subsequent analysis. These types of files are rarely shared or uploaded to an open-source framework (e.g., MorphoSource). As with most methods, the specimens are physically required for this method, thus requiring travel or shipment.

### Digital scanning

Micro-CT scanning has increased in popularity over the past decade due to several digitization initiatives from the National Science Foundation as well as from increased accessibility of scanners and reduced costs. This method allows for visualization of internal and external structures of organisms with nondestructive techniques.

#### Benefits

In spite of initial purchasing costs, micro-CT scanners can be operated for hours at a time, and used for many years (e.g., the University of Washington's Friday Harbor Laboratories micro-CT scanner has been running for over 20 h/day for more than 6 years). The methodology of scanning and subsequent analysis requires training, but widely available online tutorials are free and open access (e.g., Bruker.com, “Scan All Fishes” documentation). After scanning is completed, analyses can be done entirely off-site and each step can be delegated or shared amongst collaborators. There are more than 65,000 open-source scans available online for analysis and this number continues to grow. MorphoSource (morphosource.org), a large 3D data repository, lists 166,000 media files, 56,700 physical objects represented, and >17,000 users (as of February 2023). The same fish specimen can be scanned a single time and researchers across the globe can have all the data available for that specimen through online repositories (e.g., MorphoSource and Open Science Framework). The same data file can be used to examine different morphological parts of the fish, without necessarily having physical access the specimen. With these open-source repositories, not only researchers, but also the public are capable of interacting with 3D specimen data (e.g., Keklikoglou et al. 2016).

Unlike the other methods listed in this paper, these segmented surface models can then be 3D printed by scientists for research or for teaching or by hobbyists (Walker and Humphries 2019). This step is imperative for certain morphological comparison; 3D printing can help scientists and the general public visualize incredibly small characteristics at much larger scales than biologically normal; incredibly large morphologies can also be printed at small scales for transportation or tactile examination. The idea of holding the morphological surface in one's hands is a fantastic resource for a comparative anatomist and those interested in the biomechanical function of organisms. In biomechanical analyses, low- and high-resolution surface models can also be exported and used for novel analyses such as finite element analysis (e.g., bite forces in crocodilians; McCurry et al. 2015) or computational fluid dynamic (CFD) analysis to look at fluid flow through an anatomical structure (e.g., flow through the nose of a dinosaur; Bourke et al. (2014).

#### Downsides

Significant training in technique, software, anatomy, and measurement methods are also necessary for correct interpretation of the data to know which portions of the scan are useful. Additionally, training is necessary to understand the balance between too high of a resolution and too low; both are problematic and can result in wasted time, energy, and resources. There is also a relative subjectivity in selecting the appropriate threshold used for proper visualization of the 3D objects. While the financial costs associated with generating 3D models (photogrammetry, surface scanning, CT, or MRI) can be the highest among the methods discussed here, over time the costs have dropped substantially, and access to different scanning modalities for a wide variety of researchers has rapidly increased (Vaz et al. 2022). An additional cost includes transportation of a new specimen to and from the scanners, considering that all scanning cannot be done in-house at all natural history collections or research institutions. Although segmentation programs were a financial barrier for many researchers until recently (e.g., Mimics and Amira), free and open-source programs such as 3D Slicer (Rolfe et al. 2021) are helping to democratize the process (Hedrick et al. 2020a). After this initial barrier is cleared, micro-CT scans often result in large datasets (>6 GB each), and substantial computing power can be required to segment and render an anatomical model from the image stack. This results in an added hardware expense and potential financial barrier for students and research labs with limited funding.

### Contrast digital scanning and MRI

Several methods are included in this section, including soft-tissue staining with CT scans and MRI methods. These methods allow for the examination of both hard- and soft-tissue structures while limited destruction of specimens.

#### Benefits

Contrast-enhanced CT scanning using, for example, iodine or phosphotungstic acid, allows researchers to visualize soft tissues at the same high resolution (∼1um) as hard tissues (Gignac and Kley 2014; Gignac et al. 2016). MRI methods are ideal for studying soft tissues without the addition of potentially irreversible contrast stains, for instance, the examination of the delicate light organs of ponyfishes, which were examined *in situ* (Leiognathidae; Chakrabarty et al. 2011). These organs are made of soft tissue surrounding the esophagus that break apart easily in dissections and cannot be separated from the esophagus intact by traditional morphological approaches; however, the use of MRI technology permitted comparative analyses and the measurement of a “light organ index,” which quantified the relative volume of these features in different species. These datafiles (contrast CT and MRI) are commonly shared in open-source frameworks and can be used in subsequent analyses of morphology.

#### Downsides

In addition to the downsides listed above (see the “Digital scanning” section), the contrast agents used in this scanning method can alter the coloration of preserved specimens. Often this change is temporary, and the majority of the chemical color can be removed after scanning, but sometimes there will be a permanent change in the overall color of the specimen. This may not be ideal for rare or important specimens. The addition of contrast agents also increases the relative size of the digital files during segmentation, as more tissues can be visualized as the 3D object. As with regular scanning, this increases the need for computing power and an understanding of anatomy for correct interpretation. MRI is a lower resolution technique that does not provide the same level of detail as other scanning methods and therefore may not be as useful; although, microMRI methods are currently being developed.

### If specimens are easily accessible (no limits on sampling)

Some organisms of interest are common in natural history museum collections, the aquarium/zoo trade, or with field sampling (Poo et al. 2022). There are likely fewer restrictions imposed on these specimens by the collection staff, allowing for more destructive methods to be used to examine and quantify morphology. In other instances, rare specimens may be used sparingly with these methods to further understand their morphology, ecology, or biomechanics.

### XROMM

This method allows for examination of bone structures in the context of biomechanical motion. While less destructive than other methods in this section, it requires surgical insertion of radio-opaque beads into live organisms, requiring anesthesia and monitoring.

#### Benefits

**Fig. 2:**
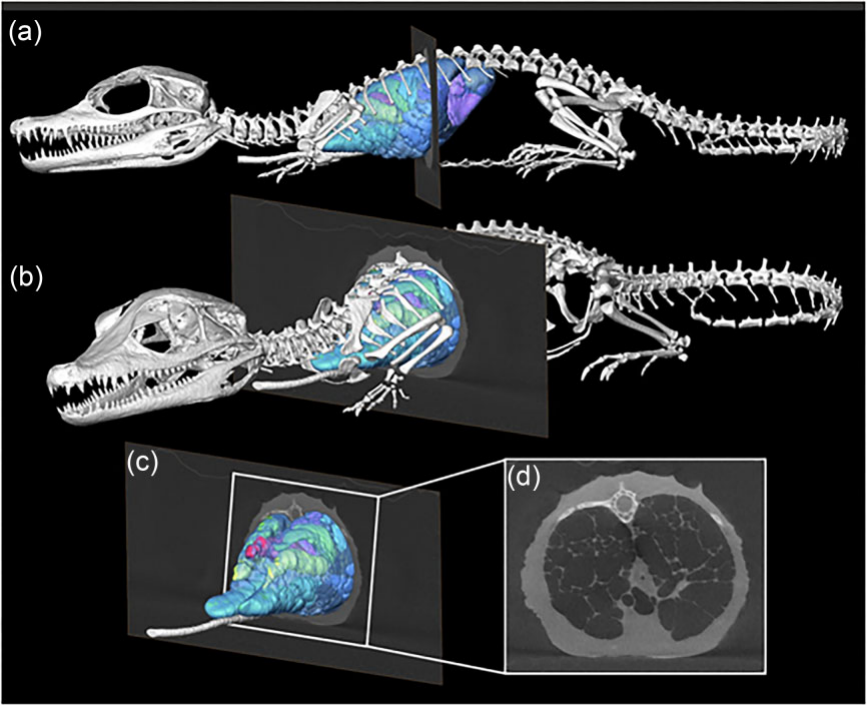
Segmented surface model of the skeleton, lung surface, and bronchial tree of a hatchling Cuvier's Dwarf Caiman (*Paleosuchus palpebrosus*) in left lateral (**a**) and craniolateral (**b**) views with a single μCT slice through the mid thorax. (**c**) Isolated μCT slice and model of the lung and bronchial tree with box indicating (**d**), which shows the axial slice of the thorax with the inflated lung parenchyma and open airways. Caiman was naturally deceased at the time of the scan, intubated and inflated. Modified from Schachner et al. (2022).

While most specimens are naturally deceased when scanned, some approaches (e.g., XROMM) also permit scanning and rendering models from live specimens demonstrating the position of the skeletal elements and internal organs in their natural living position (i.e., the size of muscles prior to desiccation, thickness and spacing of the joint cartilages, or size of the lungs during a natural apnea) (Schachner et al. 2017). This facilitates the capture of complex internal soft-tissue data *in situ*, including the cardiovascular system (Dillon et al. 2014) and negative spaces like the respiratory system that cannot be easily dissected without destroying the organ system being studied (Schachner et al. 2021) such as in reptiles (Fig. 2) who have lungs that usually collapse when excised.

#### Downsides

XROMM studies result in large datasets that must be analyzed with greater computing power (e.g., 64 GB of RAM or more). This method is also (currently) only available for use in organisms with well-ossified bones that are of a specific size. This reduces the ability to study skeletal motion in a wide range of taxa.

### Dissection

While 3D visualizations can be useful, they cannot fully replace the knowledge gained from examining actual organismal tissues. Dissection is often one of the first methods used to examine morphologies in organisms. Although it is highly destructive, it continues to be an important and standard practice for understanding anatomy and performing comparative studies.

#### Benefits

Gross dissections allow investigators to visualize morphological traits within their local organismal context. Dissections provide unique information on the material composition and ultrastructure of organs, tissues, cells and extracellular matrices, and how they connect and interact. For example, ossifications that occur within membranes (e.g., vertebrate skull roofing bones) have different ontogenetic allometries than do bones that preform within cartilages (e.g., tetrapod long bones). Such tissue specific differences are not visible in the 2D pixels or 3D voxels of most digital scanning methodologies. This kind of tissue-specific knowledge informs the investigator about variation in all tissues, for example, nervous, muscle, vasculature, etc. All anatomical investigations must begin by examining real tissues under a microscope. Digital scans complement studies of actual tissues by expanding the scope of study to the much larger sample sizes required in the study of natural populations.

#### Downsides

The main cost of dissection is that specimens are irreversibly altered, degraded, or damaged during data collection, and therefore may not be available for future work or additional studies. Although less expensive than digital scanning methods, these studies can also require expensive machinery (microscopes, cameras, etc.) and time to train the users on morphological identification, dissection techniques, and interpretation of data. Traditionally, these data from gross dissections were only disseminated in 2D as photographs or illustrations when shared or added to manuscripts. While useful for visualizing and publications, these data must be digitized for any subsequent analyses.

### Clearing and staining

In this process, specimens are rendered semi-transparent by partial digestion in the digestive enzyme Trypsin and storage in concentrated Glycerin, and targeted tissues rendered visible by the use of stable and nontoxic dyes, usually Alizarin Red for bones, Alycian Blue for cartilage, and Sudan Black for lipids such as the myelinated nerves. This is a common method for examining the internal characteristics of specimens and highly valuable for visualizing specific nonbony tissue like cartilage, ligaments, and muscle (particularly in individuals with small body sizes of 1–10 cm in total length).

#### Benefits

With this method, one can visualize the depth and layering of morphological features, facilitating simultaneous visualization of different tissue types. Two-dimensional photographs and line drawings can be taken of these specimens, for later use in linear or geometric morphometrics. It is also possible to visualize the connections between structures and the relative amount of soft tissue in the organism. This method is fairly affordable, with the main costs being the chemicals used to remove the outer tissue layers of some organisms (e.g., amniotes). These organisms can also be preserved for many years and used in subsequent analyses.

#### Downsides

Clearing and staining methods are irreversible and permanently alter the specimen. This prevents the specimen from being used in future studies (e.g., dissection, histology, digital scanning, etc.). Shrinkage of specimens occurs with all fixation techniques, including clearing and staining and histology preparation. Many specimens are also prohibitively large (most cleared and stained organisms are no more than 30 cm) and cannot be properly “cleared” with current techniques relegating this method to juveniles or small individuals of most larger species.

### Histology

Histology uses chemicals, sectioning, and microscopes to visualize the cellular- and sub-cellular level traits of a biological structure.

#### Benefits

Histological approaches offer microscopic views of organismal traits that cannot be visualized without magnification. Histochemical stains can provide concrete information on tissue types at the cellular level not available via other methods (although digital scanning has made progress in the past, see Zehbe et al. 2010). This method offers insights into tissue types and the data can be used for linear measurements as well as qualitative descriptions. Histological analysis is sometimes required to validate interpretations from micro-CT scans. Even the highest resolution scan data will not unequivocally demonstrate the morphology of a tissue that is one cell layer thick, like the air sacs of bird lungs. Biological interpretation of micro-CT images often requires knowledge of tissue types (e.g., bone that performs in cartilage, tendon, or membrane) only available from histological preparations, which improves homology assessment in coding characters for phylogenetic analysis and placing landmarks in GM analysis. Pairing histology with 3D digital models can result in highly rigorous morphological analyses. New methods of morphological examination combine computer-generated data (AI) with histological studies. These techniques allow users to quantify tissue variation across a large sample of specimens or produce 3D renderings reconstructed from histological slides (Shmatko et al. 2022).

#### Downsides

The main cost of histology is that specimens are irreversibly altered, degraded, or damaged during data collection, and therefore may not be available for future work or additional studies. Similar to digital scanning methods, this method can also require expensive machinery (microscopes, microtomes, etc.) and time to train the users on specimen preparation, morphological identification, histological techniques, and interpretation of data. These techniques are also costly in time spent preparing and imaging specimens. Data generated through these traditional methods are often maintained as microscope slides and whole-mount cleared-and-stained preparations, and disseminated mainly in the form of 2D photographs and scientific illustrations. Training largely involves one-on-one tutorials in illustration, photography, and graphic design. Documenting traditional morphological data strongly depends on personal illustration skills and other self-acquired knowledge, which can potentially lead to large inter-observer bias.

Histological slides and photographs can be sent physically or digitally, but the availability of data is generally limited to those who are willing to share the physical specimens for examination.

### SEM and TEM

SEM and TEM use electrons instead of light to form images through a microscope. This allows for high-resolution visualization of tissue and cell structure in organisms using small, dissected portions of the specimen.

#### Benefits

Methods like SEM and TEM can increase the understanding of tissue- and cellular-level morphologies by allowing examination of resolutions far above many of the aforementioned techniques. With this method one can view the surface of a structure, identify cell and tissue types, and view damage to those areas of the organism. This method is highly useful for qualitative studies and linear measurements, among other types of analyses.

#### Downsides

SEM and TEM, like micro-CT scanners, have large up-front costs for equipment and require long-term institutional support. These scanners cost hundreds of thousands of dollars and have not seen the same relative decrease in cost as digital scanners. These methods also require destruction of samples for visualization, meaning researchers either need to travel or ship specimens for their studies. The specimens do not remain whole after this technique, and therefore cannot always be used for subsequent studies. While photographs of the samples may be shared, the samples themselves can be carefully shared with shipping or with access at natural history museums. These methods are highly reliant on user training and knowledge.

## Morphological assessments

### Qualitative descriptions/diagnoses

One advantage of qualitative morphological descriptions is the capacity to discuss and compare physical traits even when they are transformed by natural processes. Fossils are often heavily distorted through diagenetics and taphonomic processes. Morphological descriptions can denote nonbiological fossil deformations, while shape analyses (whether linear, image-based, or 3D) are not able to easily account for them (Hedrick et al. 2019b; Kammerer et al. 2020). These methods are also incredibly important for taxonomic and comparative studies of traits that are not easily quantifiable.

### Quantitative methods

#### Linear morphometrics

Linear and image-based morphometrics are both relatively inexpensive methods that rely on measuring physical voucher specimens or taking digital photographs of those specimens. Linear morphometric data collection can be conducted quickly and cheaply in comparison with either 2D or 3D geometric morphometric data collection. Depending on the question that is being asked, simple length measurements, such as snout–vent length or appendicular element lengths may be all that is required to address a given hypothesis. Linear morphometric analyses are commonly used to assess topics, such as ontogenetic allometric changes with regard to locomotor or postural shifts (e.g., Hedrick et al. 2014), evolutionary allometry and heterochrony (Klingenberg 1998; Voje et al. 2014), and asymmetry (Didde and Rivera 2019). In the time required to photograph specimens for 2D geometric morphometric image-based analyses or collect scans for 3D geometric morphometric surface-based analyses, it is possible to generate a massive dataset of linear measurements. In large macroevolutionary studies, taxonomic breadth may be more important to a research question than the more precise data generated through geometric morphometrics. In these cases, linear morphometrics has a valuable place in comparative morphology.

In the case of linear morphometrics of bony tissues, resultant data no longer retain the original shape of the specimen, and thus results can be difficult to interpret compared with image-based and scan-based geometric morphometric analyses (Adams et al. 2004; Zelditch et al. 2012). Therefore, capturing the shape of a skull or other complex shapes is likely best left to either 2D or 3D geometric morphometric methods. Although 2D geometric morphometrics retains specimen shape, image-based 2D geometric morphometrics similarly has issues with highly 3D shapes where structures are flattened into *x* and *y* coordinates, losing any variation along the *z*-axis (Buser et al. 2018; Hedrick et al. 2019c; Cardini and Chiapelli 2020; Wasiljew et al. 2020). As a result, both linear and image-based methods result in reduced shape information compared with data collected using 3D imaging.

#### Geometric morphometrics

**Fig. 3.**
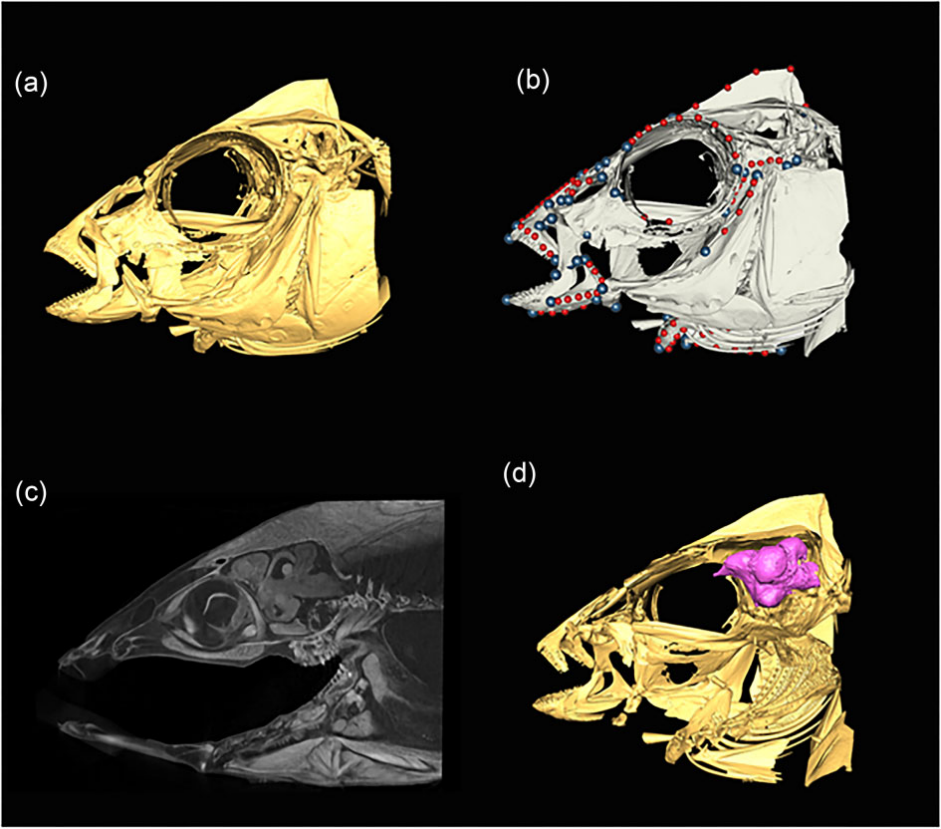
Graphical representations of the skull of *Placidochromis subocularis* (Cichlidae) illustrating various utilities of micro-CT scanning, ranging from 3D visualization (**a**), 3D geometric morphometrics (**b**, circles represent landmarks) and iodine-contrast enhanced staining (**c**, sagittal view) to highlight and isolate soft tissues like the brain (**d**, in pink).

Although linear morphometrics is a valuable method for examining a variety of different morphological questions, image-based 2D geometric morphometrics can enhance the resolution of data in many analyses (Schmieder et al. 2015). Geometric morphometrics generally is a method that allows holistic shape analysis and preserves shape relationships throughout analyses (Zelditch et al. 2012). This is in contrast to linear morphometrics, which aggregates individual length measurements into large tables where one value is not intrinsically connected to another value. This benefit has been tremendous in comparative morphology and evolutionary biology and has been termed the “Geometric Morphometric Revolution” (Rohlf and Marcus 1993; Adams et al. 2004). As such, geometric morphometric methods have spread into numerous areas of biology, including systematics, comparative morphology, evolutionary ecology, and neurobiology, among many others (Vaz et al. 2022; Fig. 3).

Compared with linear and image-based morphometric methods, 3D geometric morphometrics enables researchers to fully capture the shape of a specimen without projecting a 3D specimen into two dimensions. This provides a more realistic approximation of specimen shape, especially in complex structures (e.g., Hedrick et al. 2019c; Evans et al. 2021). However, a limitation of geometric morphometrics is comparing traits among taxa with derived shapes, because all landmarks must be present in all specimens in a given analysis. Unlike 2D geometric morphometric methods, 3D geometric morphometrics also allows for the implementation of pseudolandmarking, which can fully capture specimen shape rather than interpolating shape differences between homologous landmarks (Boyer et al. 2015; Goswami et al. 2019; Hedrick et al. 2020b) and automated landmarking, which reduces the time required to landmark large datasets (Porto et al. 2021).

## Conclusions

Newly available scanning methods permit comparisons and analyses that not possible using older, more traditional methods. However, given the limitations of each individual method described above, the best approach for studying morphology is to combine complementary techniques. New methods and faster computers, including supercomputer clusters and computational neural networks, now allow users to include many more variables (e.g., highvolume geometric morphometric data) and compare across a wide array of species and groups. These methods require additional training (and knowledge of open-source programs like R), but have dramatically increased the scope and strength of comparative morphological studies. As with many areas of science, an integrative approach to morphological studies will result in the most comprehensive examination of biological taxa. Each method has costs and benefits with no method being the perfect method directly replacing another. Integrative biologists make use of all of the methods we present from primary dissection through to synchrotron-based scanning. Matching the best method to the research question of interest in light of the costs and benefits of each individual method will facilitate comparative morphological research for years to come. Thus, we argue that the costs and benefits of all methods mentioned here promote the idea of integrative research methods rather than discounting one method over another. For instance, scanning methods complemented with the use of dry skeletons and X-ray images can allow a fuller investigation of anatomical organization than any single method.

We anticipate future studies of anatomy will integrate many or all of the aforementioned techniques for investigating the physical traits of organisms propelling the future of comparative morphology forward.
